# Synthesis, Characterization and Biological Evaluation of Some Quinoxaline Derivatives: A Promising and Potent New Class of Antitumor and Antimicrobial Agents 

**DOI:** 10.3390/molecules201119655

**Published:** 2015-11-03

**Authors:** Aisha R. Al-Marhabi, Hebat-Allah S. Abbas, Yousry A. Ammar

**Affiliations:** 1 Department of Chemistry, University College in Qunfudah, Umm Al Qura University, Qunfudah 21955, Saudi Arabia; marhaby2010@hotmail.com; 2 Department of Chemistry, Faculty of Science, King Khalid University, Abha 9004, Saudi Arabia; 3 Department of Photochemistry, National Research Centre, Dokki 12622, Egypt; 4 Department of Chemistry, Faculty of Science, Al-Azhar University, Cairo 11884, Egypt; yossry@yahoo.com

**Keywords:** quinoxaline, tetrazoloquinoxaline, 1,2,4-triazoloquinoxaline, 1,2,4-triazino-quinoxaline, anticancer agents, antimicrobial agents

## Abstract

In continuation of our endeavor towards the development of potent and effective anticancer and antimicrobial agents; the present work deals with the synthesis of some novel tetrazolo[1,5-*a*]quinoxalines, *N*-pyrazoloquinoxalines, the corresponding Schiff bases, 1,2,4-triazinoquinoxalines and 1,2,4-triazoloquinoxalines. These compounds were synthesized via the reaction of the key intermediate hydrazinoquinoxalines with various reagents and evaluated for anticancer and antimicrobial activity. The results indicated that tetrazolo[1,5-*a*]quinoxaline derivatives showed the best result, with the highest inhibitory effects towards the three tested tumor cell lines, which were higher than that of the reference doxorubicin and these compounds were non-cytotoxic to normal cells (IC_50_ values > 100 μg/mL). Also, most of synthesized compounds exhibited the highest degrees of inhibition against the tested strains of Gram positive and negative bacteria, so tetrazolo[1,5-*a*]quinoxaline derivatives show dual activity as anticancer and antimicrobial agents.

## 1. Introduction

Cancer is one of the most serious clinical problems, particularly in developed countries, despite advances in biomedical research and technology. According to the World Health Organization (WHO), the incidence of this disease is about 6 million cases per year [1,2,3]. Cancer cannot be defined as a single disease, but rather a collection of diseases [4]. Accordingly, we need to design new compounds having anticancer and antimicrobial activity at the same time. Quinoxaline derivatives display a broad spectrum of biological activities including antimicrobial [5,6,7], antiviral [8], antiinflammatory [9,10], anticancer [11,12,13,14], antimalarial [15], antitubercular, antileishmanial and kinase inhibitors [16,17,18,19]. Some quinoxaline derivatives have a cytotoxic effect on human cancer cell lines [20] and they constitute useful intermediates in organic synthesis and medicinal chemistry [21,22,23,24]. In addition, quinoxaline moiety constitutes part of the chemical structure of various antibiotics such as echinomycin, levomycin and actinoleutin that are known to inhibit the growth of Gram positive bacteria [25]. Outside medicine quinoxaline derivatives have also found applications in efficient electron luminescent materials [26], organic semiconductors [27], dyes [28], chemically controllable switches [29], building blocks for the synthesis of anion receptors, cavitands and dehydoannulenes [30,31]. They also serve as useful rigid subunits in macrocyclic receptors in molecular recognition [32]. Hydrazine quinoxalines and their cyclic analogues were reported as antimicrobial, anticonvulsant, analgesic, antiinflammatory, antiplatelet, antitubercular and antitumor [33] agents. Tetrazoloquinoxalines have been reported for their antibacterial, antifungal or algicidal [34] activities. The above findings gave us the idea for the design and synthesis of new compounds containing bromoquinoxaline derivatives and their *in vitro* evaluation for antitumor and antimicrobial activity.

## 2. Results and Discussion

### 2.1. Chemistry

**Scheme 1:**
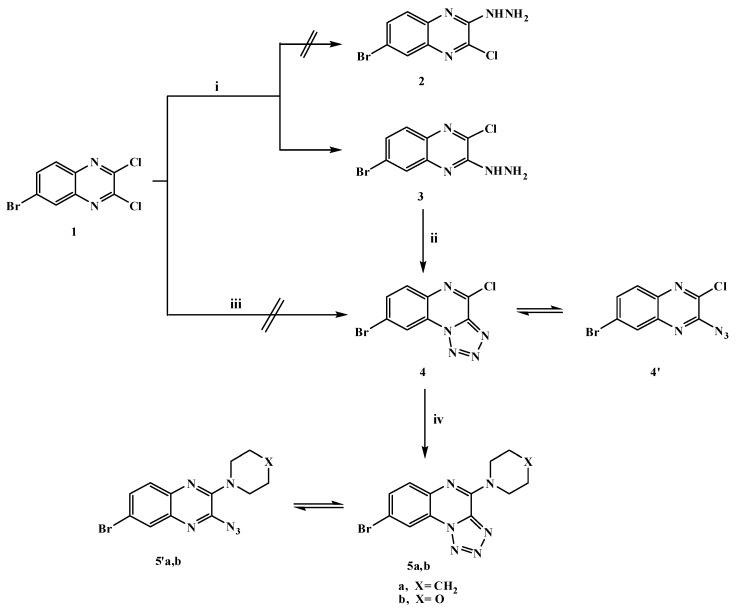
General methods for the preparation of compounds **3**–**5a**,**b**. *Reagents and conditions*: (i) hydrazine hydrate 90% (1.5 mol)/EtOH/0 °C, stirring; (ii) NaNO_2_/AcOH/H_2_O/0–5 °C, stirring; (iii) NaN_3_/EtOH, reflux; and (iv) piperidine or morpholine/CH_3_CN/K_2_CO_3_, reflux.

**Scheme 2:**
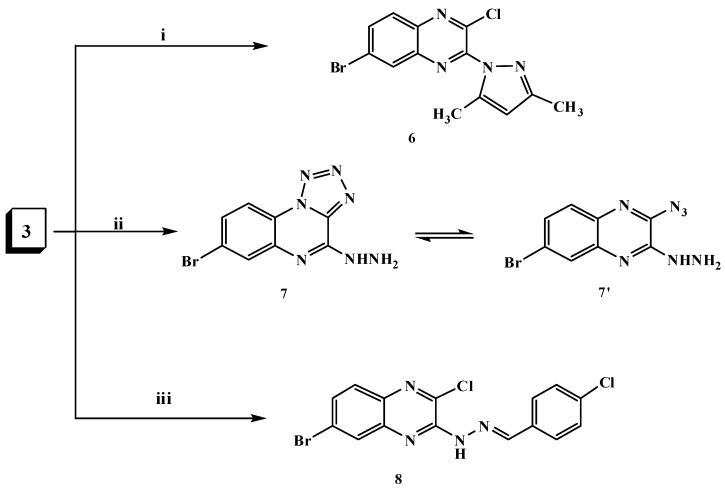
General methods for the preparation of compounds **6**–**8**. *Reagents and conditions*: (i) Acetylacetone/piperidine/EtOH,reflux; (ii) NaN_3_/EtOH,reflux; and (iii) *p*-chlorobenzaldehyde/EtOH/AcOH, reflux.

**Scheme 3:**
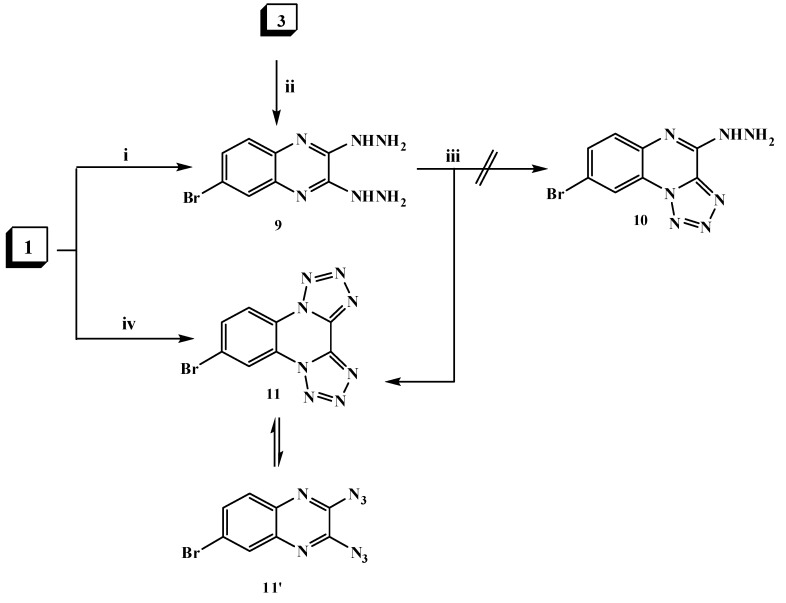
General methods for the preparation of compounds **9**–**11**. *Reagents and conditions*: (i) hydrazine hydrate 90% (2.5 mol)/EtOH, reflux; (ii) hydrazine hydrate 90% (1.5 mol)/EtOH, reflux; (iii) NaNO_2_ AcOH/H_2_O/0–5 °C; stirring and (iv) NaN_3_ (2 mol)/EtOH, reflux.

**Scheme 4:**
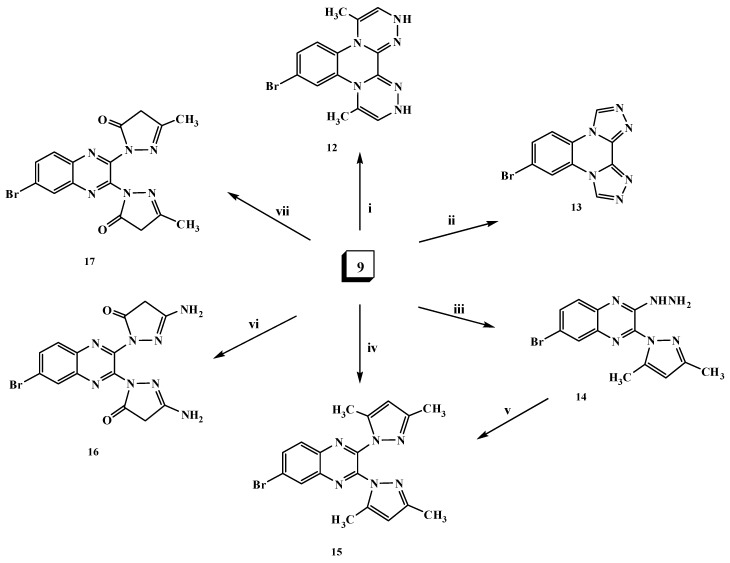
General methods for the preparation of compounds **12**–**17**. *Reagents and conditions*: (i) Chloroacetone/DMF/AcOH, reflux; (ii) triethyl orthoformate/EtOH/AcOH, reflux; (iii) acetylacetone (1 mol)/EtOH/piperidine, reflux; (iv) acetylacetone (2 mol)/EtOH/NaOEt, reflux; (v) acetylacetone (1 mol)/EtOH/piperidine, reflux; (vi) ethyl cyanoacetate (2 mol)/EtOH/NaOEt, reflux; and (vii) ethyl acetoacetate (2 mol)/EtOH/NaOEt, reflux.

The synthesis of the target compounds is depicted in Scheme 1, Scheme 2, Scheme 3 and Scheme 4. The starting compound, 6-bromo-2,3-dicloroquinoxaline (**1**) was prepared in good yield as described in the literature [35,36,37] by the reaction of 4-bromo-*o*-phenylenediamine with oxalic acid in hydrochloric acid to give 6-bromo-1,4-dihydroquinoxaline-2,3-dione, followed by the chlorination of the latter compound with phosphorus oxychloride in the presence of DMF as the catalyst. Dichloroquinoxaline derivative **1** is a good precursor for the synthesis of different new heterocyclic compounds with the hope of obtaining some biologically active substituted quinoxaline derivatives. Its reactivity toward many types of nucleophilic reagents enables studying the effect of bromine atom on the reactivity of two chlorine atoms. Thus, when dichloroquinoxaline derivative **1** reacted with one mole of hydrazine hydrate in absolute ethanol, 6-bromo-2-chloro-3-hydrazinylquinoxaline (**3**) was produced, however our attempts to synthesize 6-bromo-3-chloro-2-hydrazinylquinoxaline (**2**) failed as shown in Scheme 1. This indicated that the chlorine atom in the 3-position was more reactive than the one in the 2-position and presumed to be preferentially substituted from the consideration of the (-R) effect of the bromine atom in compound **1**, which is the factor responsible for the formation of compound **3**. The structure of compound **3** was evidenced by its spectroscopic and elemental analysis data. Its IR spectrum showed strong bands at 3415, 3250 and 3146 cm^−1^ indicating the presence of the HNNH_2_ group and its ^1^H-NMR spectrum exhibited the characteristic a broad D_2_O exchangeable singlets at δ 5.00 and 9.16 ppm due to the NH_2_ and NH protons, respectively. 

The hydrazine analogue **3** was used as the key compound to facilitate the synthesis of some bioactive compounds. Thus, treatment of 3-hydrazinyl derivative **3** with sodium nitrite in acetic acid at 0 °C afforded the tetrazoloquinoxaline **4** in 75% yield, but it was not formed via the reaction of dichloroquinoxaline derivative **1** with one mole of sodium azide as shown in Scheme 1. The new ring system of tetrazolo derivative **4** was in equilibrium with the corresponding 3-azido tautomer **4′** [38], what was confirmed by the disappearance of the HNNH_2_ bands and a characteristic N_3_ group absorption band at 2242 cm^−1^ in the IR spectrum; ^1^H-, ^13^C-NMR and mass spectra were in accordance with its structure. Nucleophilic replacement of the chlorine atom of tetrazolo derivative **4** was performed by refluxing with cyclic secondary amines, namely pipridine or morpholine in acetonitrile and anhydrous K_2_CO_3_ furnishing the corresponding 8-bromo-(4-substituted amino)tetrazolo[1,5-*a*]quinoxalines **5a**,**b** (Scheme 1). The ring system in derivatives **5a**,**b** was in tautomeric equilibrium with the azido derivatives **5′a**,**b**. The analytical and spectral data were in agreement with the proposed structures. Considering the IR spectrum of compound **5b** as an example, the bands that appear at 2925, 2865 cm^−1^ represent aliphatic C-H and the band at 2241 cm^−1^ the N_3_ group. ^1^H-NMR spectrum showed multiple signals at δ 3.82 and 4.28 ppm corresponding to aliphatic 2N-CH_2_ and 2O-CH_2_ protons of morpholine, respectively. The *N*-pyrazolo derivative **6** was obtained in good yield when compound **3** was allowed to react with a active methylene compound (acetylacetone) in ethanol containing a few drops of piperidine as shown in Scheme 2. The structure of compound **6** was elucidated on the basis of its spectral and analytical data. The IR spectrum revealed the absence of the characteristic bands of a HNNH_2_ group and its ^1^H-NMR spectrum revealed singlets at δ 2.01, 2.20 and 5.98 ppm due to the presence of 2 × CH_3_ protons and a C*H*-pyrazole proton, respectively. The ^13^C-NMR spectral data displayed characteristic signals at 13.85 ppm and 14.02 ppm for the two methyls and 107.83 ppm for *C*H-pyrazole while its mass spectrum showed a molecular ion peak at *m*/*z* 336 (M^+^,44%), which was in agreement with the proposed structure. In addition, the reaction of compound **3** with sodium azide in ethanol led to the formation in good yield of hydrazinyltetrazoloquinoxaline **7**, which was found to be in tautomeric equilibrium with the azido derivative **7′** (Scheme 2). The structure of compound **7** was elucidated on the basis of elemental analysis and spectral data which were an agreement with the proposed structure. Furthermore, to get a new Schiff base that was expected to be biologically active, heating 3-hydrazinyl derivative **3** with *p*-chlorobenzaldehyde in ethanol containing a few drops of glacial acetic acid furnished the corresponding Schiff base **8** as shown in Scheme 2. The structure was characterized by the disappearance of the NH_2_ bands in its IR spectrum, ^1^H-NMR spectrum showed a singlet at δ 8.64 ppm due to the presence of a C*H*=N- azomethine proton.

6-Bromo-2,3-dihydrazinylquinoxaline (**9**) was formed via two routes. First, via hydrazinolysis of 2,3-dichloroquinoxaline derivative **1** with two moles of hydrazine hydrate by refluxing in ethanol. The second route involveh the reaction of 3-hydrazinyl derivative **3** with another mole of hydrazine hydrate (Scheme 3). The structure of compound **9** was elucidated on the basis of elemental analysis and spectral data and its mass spectrum showed a molecular ion peak at *m*/*z* 268 (M^+^, 43%).

The hydrazine analogue **9** was used as the key intermediate for the synthesis of some different heterocyclic compounds. Thus, treatment of dihydrazinylquinoxaline derivative **9** with one mole of nitrous acid at 0 °C led to the formation of the ditetrazoloquinoxaline **11** in good yield, while mono-tetrazolo[1,5-*a*]quinoxaline **10** was not obtained. Compound **11** was furnished via treatment of the 2,3-dichloroquinoxaline derivative **1** with two moles of sodium azide in ethanol as shown in Scheme 3. Compound **11** was found to be in tautomeric equilibrium with the 2,3-diazido derivative **11′**. Its IR spectrum revealed the absence of the characteristic HNNH_2_ group band and its mass spectrum showed a molecular ion peak at *m*/*z* 290 (M^+^; 7%). Heating of compound **9** with an α-haloketone, namely chloroacetone, in the presence of a few drops of acetic acid in DMF resulted in the formation of the corresponding di-[1,2,4]triazinoquinoxaline derivative **12** as shown in Scheme 4. The structure of compound **12** was confirmed by spectral data. The ^1^H-NMR spectrum revealed a singlet at δ 9.3 ppm due to C*H*-triazine and a broad singlet (D_2_O exchangeable) at δ 10.75 ppm due to the NH groups.

The formation of the di-[1,2,4]triazoloquinoxaline derivative **13** was achieved upon the reaction of compound **9** with triethyl orthoformate in ethanol in the presence of a few drops of acetic acid (Scheme 4). The ^1^H-NMR spectrum of compound **13** revealed the disappearance of the HNNH_2_ signals and appearance of a singlet signal at δ 8.55 ppm due to the C*H-*triazole proton. Furthermore, in the reaction of compound **9** with one mole of acetylacetone in ethanol containing a few drops of piperidine, the *N*-pyrazolo derivative **14** was produced (Scheme 4). The IR spectrum of compound **14** showed the characteristic HNNH_2_ group bands at 3448, 3277 and 3191 cm^−1^, and its ^1^H-NMR spectrum revealed singlets at δ 2.01 and 2.20 ppm due to the presence of 2 × CH_3_ protons and a singlet at δ 6.05 ppm due to the presence of the C*H*-pyrazole proton. The ^13^C-NMR spectral data displayed characteristic signals at δ 13.86, 13.94 for the 2two methyl groups and 109.51 ppm for *C*H-pyrazole. On the other hand, upon heating the dihydrazino compound **9** with two moles of an active methylene (β-diketone) compound, namely acetylacetone or ethyl cyanoacetate or/and ethyl acetoacetate, in ethanolic sodium ethoxide solution, the *N*-pyrazolo derivatives **15**–**17** were produced (Scheme 4). For example, the structure of compound **15** was confirmed by the absence of the characteristic HNNH_2_ group bands in its IR spectrum. Its structure was evidenced by its ^1^H- and ^13^C-NMR, mass spectra and elemental analysis data. Moreover, compound **15** was confirmed chemically by the reaction of the *N*-pyrazolo derivative **14** with another mole of acetylacetone (Scheme 4).

### 2.2. Pharmacological Screening

#### 2.2.1. *In Vitro* Anticancer Screening

The *in vitro* cytotoxic activity of the newly synthesized compounds against human breast cell line (MCF7), non-small cell lung cancer NCIH460, CNS cancer SF-268 and WI 38 (normal fibroblast cells) were evaluated using doxorubicin as the reference drug according to the method reported by Skehan *et al.* [39]. The IC_50_ of the synthesized compounds compared to the reference drug is shown in Table 1.

**Table 1:** Cytotoxic activity in (IC_50_, μg/mL) by the newly synthesized compounds against human cancer cell lines and normal cells.

Compound No.	IC_50_ (μg/mL)			
	MCF-7	NCI-H460	SF-268	WI 38
**3**	36.02 ± 7.33 ^c^	26.74 ± 2.18 ^b^	30.64 ± 2.39 ^b^	32.16 ± 6.54
**4**	0.02 ± 0.006 ^a^	0.01 ± 0.004 ^a^	0.06 ± 0.003 ^a^	non-cytotoxic
**5a**	0.01 ± 0.001 ^a^	0.02 ± 0.006 ^a^	0.02 ± 0.008 ^a^	non-cytotoxic
**5b**	0.02 ± 0.002 ^a^	0.01 ± 0.002 ^a^	0.06 ± 0.008 ^a^	non-cytotoxic
**6**	22.41 ± 10.4 ^b^	30.48 ± 10.8 ^b^	26.51 ± 2.87 ^b^	28.25 ± 0.87
**7**	26.4 ± 2.10 ^b^	12.42 ± 3.01 ^b^	10.63 ± 2.83 ^b^	non-cytotoxic
**8**	42.16 ± 2.46 ^c^	26.60 ± 2.63 ^b^	35.32 ± 12.81 ^c^	10.59 ± 5.51
**9**	37.07 ± 7.34 ^c^	16.37 ± 2.32 ^b^	38.94 ± 2.63 ^c^	30.62 ± 6.21
**11**	1.18 ± 1.03 ^a^	2.83 ± 0.53 ^a^	2.86 ± 4.92 ^a^	56.85 ± 4.05
**12**	35.22 ± 4.18 ^c^	34.03 ± 6.05 ^c^	22.10 ± 2.81 ^b^	22.97 ± 8.2
**13**	37.64 ± 6.72 ^c^	36.05 ± 5.23 ^c^	29.35 ± 7.01 ^c^	18.62 ± 1.21
**14**	30.32 ± 3.86 ^b^	38.32 ± 2.35 ^c^	42.06 ± 5.58 ^c^	58.70 ± 8.65
**15**	28.42 ± 5.80 ^b^	22.73 ± 8.12 ^b^	30.24 ± 2.04 ^b^	18.16 ± 4.03
**16**	12.82 ± 1.46 ^b^	22.95 ± 0.46 ^b^	49.85 ± 8.64 ^c^	30.03 ± 2.36
**17**	21.23 ± 0.14 ^b^	15.81 ± 0.10 ^b^	21.33 ± 2.12 ^b^	21.40 ± 2.02
DMSO	0	0	0	0
Doxorubicin	0.04 ± 0.008	0.09 ± 0.008	0.09 ± 0.007	non-cytotoxic

MCF-7 (breast adenocarcinoma); NCI-H460 (non-small cell lung cancer); SF-268 (CNS cancer); WI 38 (Normal fibroblast cells); Doxorubicin (anticancer positive control); DMSO (solvent, negative control); ^a^ highly active, ^b^ moderately active, ^c^ weakly active.

From the results presented in Table 1, it is evident that some of the compounds were active against the three human cancer cell lines. Compounds **4**, **5a** and **5b** displayed high cytotoxic activity against the tested cell lines (most of IC_50_ the values ranged from 0.01 ± 0.001 to 0.06 ± 0.008 μg/mL) and these compounds were non-cytotoxic on the normal cells (IC_50_ values >100 μg/mL) and exhibited better cytotoxicity against most of cancer cell lines than doxorubicin. Moreover, compound **11** exhibited high growth inhibitory activity on the various cancer panel cell lines (IC_50_ values ranged from 1.18 ± 1.03 to 2.86 ± 4.92 μg/mL) with high cytotoxicity on normal cells (IC_50_ values ranged from 56.85 ± 4.05 μg/mL). In addition, the compounds **3**, **6**–**9** and **12**–**17**, exhibited a moderate to weak cytotoxicity against all cancer cell lines (IC_50_ values ranged from 10.63 ± 2.83 to 42.16 ± 2.46 μg/mL) with cytotoxic effects on the human normal cell (IC_50_ values ranged from non-cytotoxic to 58.70 ± 8.65 μg/mL) compared with doxorubicin.

#### 2.2.2. Antimicrobial Activity

All the newly synthesized compounds were evaluated for their *in vitro* antimicrobial activities against *Bacillus subtilis, Staphylococcus aureus* and *Streptococcus faecalis* as examples of Gram positive bacteria, *Escherichia coli*, *Neisseria gonorrhoeae*, *Pseudomonas aeruginosa* and *Salmonella typhimurium* as examples of Gram negative bacteria and *Aspergillus flavus*, *Aspergillus fumigatus* and *Candida albicans* as examples of fungal strains. 

The disk diffusion method was used for determination of the antimicrobial activity using ampicillin, gentamicin and amphotericin B as reference drugs. The results were recorded for each compounds as the average diameter as inhibition zones (IZ) of bacterial or fungal growth around the discs in mm as shown in Table 2. Minimum inhibitory concentration (MIC) measurements were determined for the compounds as shown in Table 3.

##### Antibacterial Activity

According to the obtained results (Table 2 and Table 3), it is clear that most compounds showed significant activity against all types of bacterial strains. Compounds **4**, **5a** and **9**–**13** were found to be highly active against Gram positive and negative bacteria (IZ = 17–31 mm and MIC values ranged from 0.98 to >125 μg/mL).

3-Hydrazinyl quinoxaline **3** showed moderate activity towards Gram positive and negative bacteria (IZ = 13–15 mm and MIC values ranged from 0.98 to 125 μg/mL). Likewise, compounds **5b** and **14**–**17** showed weak activity against most strains of Gram positive and negative bacteria (IZ = 9–12 mm and MIC values ranged from 1.95 to 125 μg/mL).

##### Antifungal Activity

From the IZ data recorded in Table 2 and Table 3 most of the compounds showed no significant antifungal activity, except compounds **4**, **5a**, **5b** and **9**. Compound **4** displayed high antifungal activity towards *C. albicans* (IZ = 20 mm and MIC = 3.9 μg/mL) and it was moderately active against *A. flavus* (IZ = 13 mm), while the compounds **5a**, **5b** and **9** showed weak activity against *A. fumigatus* and *C. albicans* (IZ = 5–13 mm and MIC values ranged from 0.98 to 62.5 μg/mL).

**Table 2:** Inhibition zone in (mm) as a criterion of antimicrobial activity of the newly synthesized compounds.

Compound No.	Inhibition Zone Diameter (IZ) (mm)									
	Gram Positive Bacteria			Gram Negative Bacteria				Fungi		
	*Bacillus subtilis*	*Staphylococcus aureus*	*Streptococcus faecalis*	*Escherichia coli*	*Neisseria gonorrhoeae*	*Pseudomonas aeruginosa*	*Salmonella typhimurium*	*Aspergillus flavus*	*Aspergillus fumigatus*	*Candida albicans*
**3**	15	14	14	13	14	14	13	R	R	R
**4**	20	26	18	17	21	21	17	13	10	20
**5a**	13	11	9	13	R	11	14	R	12	12
**5b**	17	17	11	11	R	10	13	R	12	9
**6**	11	9	11	11	11	9	8	R	R	R
**7**	11	10	11	10	10	10	9	R	R	R
**8**	9	9	10	11	9	9	7	R	R	R
**9**	28	27	27	29	28	31	17	R	5	11
**11**	19	17	15	15	R	14	18	R	R	R
**12**	18	19	18	17	R	20	18	R	R	R
**13**	17	16	20	17	R	19	17	R	R	R
**14**	12	12	12	12	12	12	10	R	R	R
**15**	10	9	9	10	9	9	10	R	R	R
**16**	10	12	11	10	11	11	10	R	R	R
**17**	7	8	10	11	6	8	5	R	R	R
DMSO	0	0	0	0	0	0	0	0	0	0
Ampicillin	20	22	19	-	-	-	-	-	-	-
Gentamicin	-	-	-	20	18	17	23	-	-	-
Amphotericin B	-	-	-	-	-	-	-	17	23	19

Highly active (IZ ≥ 17 mm); moderately active (IZ = 13–16 mm); weakly active (IZ ≤ 12 mm); R (IZ = 0 mm); Ampicillin (antibacterial positive control for Gram positive bacteria); Gentamicin (antibacterial positive control for Gram negative bacteria); Amphotericin B (antifungal control); DMSO (solvent, negative control).

**Table 3:** Minimum inhibitory concentration (MIC) in (μg/mL) showing antimicrobial activities of the tested compounds compared with the reference drugs.

Compound No.	Minimum Inhibitory Concentration (MIC) (µg/mL)							
	Gram positive Bacteria			Gram Negative Bacteria			Fungi	
	*Bacillus subtilis*	*Staphylococcus aureus*	*Streptococcus faecalis*	*Escherichia coli*	*Pseudomonas aeruginosa*	*Salmonella typhimurium*	*Aspergillus fumigatus*	*Candida albicans*
**3**	1.95	3.9	15.63	3.9	125	1.95	-	-
**4**	0.98	3.9	0.98	3.9	>125	0.98	-	3.9
**5a**	31.25	62.5	62.5	125	125	31.25	31.25	62.5
**5b**	1.95	1.95	3.9	1.95	>125	1.95	1.95	3.9
**9**	0.98	1.95	0.98	1.95	125	0.98	0.98	0.98
**10**	0.98	0.98	0.98	3.9	125	1.95	-	-
**11**	0.98	7.81	62.5	62.5	125	7.81	-	-
**13**	1.95	7.81	62.5	7.81	125	3.9	-	-
**14**	1.95	1.95	1.95	3.9	125	1.95	-	-
**16**	1.95	1.95	31.25	3.9	125	1.95	-	-

## 3. Experimental Section

### 3.1. General Information

All melting points are uncorrected and were taken in open capillary tubes using the Stuart Digital Melting Point SMP10 (Stuart, Staffordshire, UK). Elemental analysis data (in accord with the calculated values) were performed by Vario, Elementar apparatus (Shimadzu, Kyoto, Japan). IR spectra were recorded on a Thermo Nicolet (Thermo Scientific, Madison, WI, USA) using KBr disks. The NMR spectra (500/125 MHz) were determined by using a Bruker NMR spectrometer (Rheinstetten, Muchen, Germany). Chemical shifts are expressed in δ (ppm) downfield from TMS as an internal standard. Mass spectra were recorded at 70 ev EI Ms-QP 1000 EX (Shimadzu, Kyoto, Japan). Monitoring of the reactions and checking the purity of the compounds was performed by TLC on silica gel-precoated aluminium sheets (Fluorescent indicator 254 nm, Fluka, Muchen, Germany) and the spots were detected by exposure to UV lamp at λ 254/366 nm for a few seconds or under iodine vapor. Silica gel 60 (Merck, Munchen, Germany) was used for column chromatography. Biological evaluation of the newly synthesized products was performed by a research group of the National Cancer Institute at Cairo University, Egypt.

#### 3.1.1. 6-Bromo-2-chloro-3-hydrazinylquinoxaline (**3**)

To a solution of compound **1** (0.28 g, 1 mmol) in absolute ethanol (50 mL), hydrazine hydrate 90% (0.07 mL, 1.5 mmol) was added and the reaction mixture was stirred in an ice bath at 0 °C for 2 h. After completion of the reaction, the precipitate that formed was filtered, dried and the crude product was further purified by a silica gel column chromatography (chloroform) to give the product. Yield: 61%; (red-brown powder): mp 201–203 °C; IR (KBr) ν_max_ in cm^−1^: 3415, 3250, 3146 (NH_2_, NH), 1598 (C=N); ^1^H-NMR (DMSO-*d*_6_): δ 5.00 (s, br, 2H, NH_2_; exchangeable with D_2_O), 7.37–7.79 (m, 3H, Ar-H), 9.16 (s, br, 1H, NH; exchangeable with D_2_O); ^13^C-NMR (DMSO-*d*_6_): δ 124.68, 129.67, 130.02, 134.78, 138.98, 140.60 (6Ar-C), 145.22, 145.85, (2C=N); MS (*m*/*z*), 64 (M^+^ − C_4_H_4_BrClN_3_; 100%), 272 (M^+^; 5%), 273 (M^+^ + 1; 21%), 274 (M^+^ + 2; 17%), 275 (M^+^ + 3; 3%), 276 (M^+^ + 4; 5%). Anal. Calcd. for C_8_H_6_BrClN_4_ (273.52): C, 35.13; H, 2.21; N, 20.48%. Found: C, 34.97; H, 2.45; N, 20.34%.

#### 3.1.2. 8-Bromo-4-chlorotetrazolo[1,5-*a*]quinoxaline (**4**)

A solution of sodium nitrite (0.07 g, 1 mmol) in the least amount of water was added dropwise to an ice-cold solution of compound **3** (0.27 g, 1 mmol) in acetic acid (30 mL) kept in an ice bath at 0 °C. The reaction mixture was allowed to stand overnight at room temperature, then it was poured into water (100 mL). The precipitate that formed was filtered, dried and purified by a silica gel column chromatography (chloroform) to give the product. Yield: 75%; (pale brown powder): mp 199–201 °C; IR (KBr) ν_max_ in cm^−1^: 2242 (N_3_), 1603 (C=N); ^1^H-NMR (DMSO-*d*_6_): δ 8.13–8.82 (m, 3H, Ar-H); ^13^C-NMR (DMSO-*d*_6_): δ 121.70, 125.82, 129.83, 131.82, 132.57, 133.20 (6Ar-C), 143.26, 143.74 (2C=N); MS (*m*/*z*), 63 (M^+^ − C_4_H_4_BrClN_4_; 98%), 257 (M^+^ − CN; 100%), 283 (M^+^; 9%), 284 (M^+^ + 1; 2%), 285 (M^+^ + 2; 11%), 286 (M^+^ + 3; 1%), 287 (M^+^ + 4; 3%). Anal. Calcd. for C_8_H_3_BrClN_5_ (284.50): C, 33.77; H, 1.06; N, 24.62 %. Found: C, 33.86; H, 1.29; N, 24.51%.

#### 3.1.3. General Procedure for Synthesis of 8-Bromo-4-(substituted amino)tetrazolo[1,5-*a*]quinoxaline (**5a**,**b**)

Amine (1 mmol) was dissolved in acetonitrile (50 mL). Anhydrous potassium carbonate (2.0 g) was added to the mixture, which was refluxed for 1 h, then compound **4** (0.28 g, 1 mmol) was added and the mixture was further refluxed for 2 h. After completion of the reaction, the reaction mixture was filtrered to remove the potassium carbonate, then the excess of acetonitrile was evaporated under reduced pressure and residue obtained was dried and purified by a silica gel column chromatography to give the product.

##### 8-Bromo-4-(piperidin-1-yl)tetrazolo[1,5-*a*]quinoxaline (**5a**)

The compound **5a** was obtained from the reaction of piperidine and purified by a silica gel column chromatography (chloroform as eluent). Yield: 72%; (orange powder): mp 122–123 °C; IR (KBr) ν_max_ in cm^−1^: 2934, 2850 (aliphatic C-H), 2246 (N_3_), 1598 (C=N); ^1^H-NMR (DMSO-*d*_6_): δ 1.72 (m, 6H, 3CH_2_), 3.88 (m, 4H, 2N-CH_2_), 7.59–8.39 (m, 3H, Ar-H); ^13^C-NMR (DMSO-*d*_6_): δ 22.36, 24.01, 25.60 (3CH_2_), 46.84 (2N-CH_2_), 121.93, 122.36, 126.58, 127.69, 128.62, 132.37 (6Ar-C), 139.16, 144.97 (2C=N); MS (*m*/*z*), 248 (M^+^ − piperidine; 97%), 250 (M^+^ − C_2_H_2_N_4_; 100%), 332 (M^+^; 20%), 333 (M^+^ + 1; 5%), 334 (M^+^ + 2; 20%). Anal. Calcd. for C_13_H_13_BrN_6_ (333.19): C, 46.86; H, 3.93; N, 25.22%. Found: C, 46.97; H, 4.12; N, 25.41%.

##### 8-Bromo-4-(morpholin-4-yl)tetrazolo[1,5-*a*]quinoxaline (**5b**)

Compound **5b** was obtained from the reaction of morpholine and purified by a silica gel column chromatography (5:0.2, chloroform/methanol as eluent). Yield: 84%; (orange powder): mp 144–146 °C; IR (KBr) ν_max_ in cm^−1^: 2925, 2865 (aliphatic C-H), 2241 (N_3_), 1602 (C=N); ^1^H-NMR (DMSO-*d*_6_): δ 3.82 (m, 4H, 2N-CH_2_), 4.28 (m, 4H, 2O-CH_2_), 7.62–8.43 (m, 3H, Ar-H); ^13^C-NMR (DMSO-*d*_6_): δ 46.30 (2N-CH_2_), 65.94, 66.33 (2O-CH_2_), 120.96, 122.56, 127.21, 127.93, 132.51, 136.16 (6Ar-C), 145.35, 145.69 (2C=N); MS (*m*/*z*), 248 (M^+^ − morpholine; 99%), 250 (M^+^ − C_2_H_4_N_4_; 100%), 334 (M^+^; 17%), 335 (M^+^ + 1; 3%), 336 (M^+^ + 2; 16%). Anal. Calcd. for C_12_H_11_BrN_6_O (335.16): C, 43.00; H, 3.31; N, 25.07%. Found: C, 43.18; H, 3.52; N, 25.30%.

#### 3.1.4. 6-Bromo-2-chloro-3-(3,5-dimethyl-1*H*-pyrazol-1-yl)quinoxaline (**6**)

To a solution of compound **3** (0.27 g, 1 mmol) in absolute ethanol (50 mL), acetylacetone (0.10 mL, 1 mmol) and few drops of piperidine were added. The reaction mixture was refluxed for 6 h. After completion of the reaction, the solvent was evaporated under reduced pressure and residue obtained was purified by a silica gel column chromatography (5:0.3, petroleum ether (60–80)/ethyl acetate as eluent) to give the product. Yield: 73%; (brown powder): mp 81–83 °C; IR (KBr) ν_max_ in cm^−1^: 2948, 2842 (aliphatic C-H), 1603 (C=N); ^1^H-NMR (DMSO-*d*_6_): δ 2.01, 2.20 (2s, 6H, 2CH_3_), 5.98 (s, 1H, C*H*-pyrazole), 8.10–8.40 (m, 3H, Ar-H); ^13^C-NMR (DMSO-*d*_6_): δ 13.85, 14.02 (2CH_3_), 107.83 (*C*H-pyrazole), 118.65, 127.67, 127.93, 129.28, 135.40, 137.41 (7Ar-C), 149.35, 149.90, 150.74 (3C=N); MS (*m*/*z*), 301 (M^+^ − Cl; 100%), 336 (M^+^; 44%), 337 (M^+^ + 1; 12%), 338 (M^+^ + 2; 58%), 339 (M^+^ + 3, 10%), 340 (M^+^ + 4; 14%). Anal. Calcd. for C_13_H_10_BrClN_4_ (337.60): C, 46.25; H, 2.99; N, 16.60%. Found: C, 46.10; H, 2.83; N, 16.49%.

#### 3.1.5. 7-Bromo-4-hydrazinyltetrazolo[1,5-*a*]quinoxaline (**7**)

To a solution of compound **3** (0.27 g, 1 mmol) in absolute ethanol (50 mL), sodium azide (0.06 g, 1 mmol) was added and the reaction mixture was refluxed for two days. After completion of the reaction, the reaction mixture was filtered, then the solvent was evaporated under reduced pressure and residue obtained was dried and crystallized from benzene to give the product. Yield: 98%; (brown powder): mp 237–239 °C; IR (KBr) ν_max_ in cm^−1^: 3380, 3355, 3180 (NH_2_, NH), 2130 (N_3_), 1594 (C=N); ^1^H-NMR (DMSO-*d*_6_): δ 4.97 (s, br, 2H, NH_2_; exchangeable with D_2_O); 7.59–8.29 (m, 3H, Ar-H), 9.84 (s, br, 1H, NH; exchangeable with D_2_O); ^13^C-NMR (DMSO-*d*_6_): δ 124.68, 129.67, 130.02, 134.78, 138.98, 140.60 (6Ar-C), 145.22, 145.85 (2C=N); MS (*m*/*z*), 130 (M^+^ − C_5_H_5_N_6_; 100%), 279 (M^+^; 4%), 280 (M^+^ + 1; 2%), 281 (M^+^ + 2; 3%). Anal. Calcd. for C_8_H_6_BrN_7_ (280.08): C, 34.31; H, 2.16; N, 35.01%. Found: C, 34.45; H, 2.14; N, 35.20%.

#### 3.1.6. 6-Bromo-2-chloro-3-[2-(4-chlorobenzylidene)hydrazinyl]quinoxaline (**8**)

A mixture of compound **3** (0.27 g, 1 mmol) and *p*-chlorobenzaldehyde (0.14 g, 1 mmol) in absolute ethanol (50 mL) and few drops of concentrated glacial acetic acid was refluxed for 3 h. After completion of the reaction, the solvent was evaporated under reduced pressure and residue obtained was purified by a silica gel column chromatography (2:1, chloroform/petroleum ether (60–80)) to give the product. Yield: 89%; (orange powder): mp 187–189 °C; IR (KBr) ν_max_ in cm^−1^: 3146 (NH), 1616 (C=N); ^1^H-NMR (DMSO-*d*_6_): δ 7.54–8.05 (m, 7H, Ar-H), 8.64 (s, br, 1H, CH=N-), 11.26 (s, br, 1H, NH; exchangeable with D_2_O); ^13^C-NMR (DMSO*-d*_6_): δ 117.66, 123.48, 128.38, 128.60, 128.73, 128.92, 129.25, 130.11, 133.45, 141.52 (12Ar-C), 145.38, 146.56 (2C=N), 156.82 (CH=N-); MS (*m*/*z*), 259 (M^+^ − C_4_H_3_Cl_2_N; 100%), 394 (M^+^; 8%), 395 (M^+^ + 1; 5%), 396 (M^+^ + 2; 12%), 397 (M^+^ + 3; 4%), 398 (M^+^ + 4; 5%). Anal. Calcd. for C_15_H_9_BrCl_2_N_4_ (396.07): C, 45.49; H, 2.29; N, 14.15%. Found: C, 45.67; H, 2.08; N, 14.02.

#### 3.1.7. 6-Bromo-2,3-dihydrazinylquinoxaline (**9**)

*Method A*: To a solution of compound **1** (0.28 g, 1 mmol) in absolute ethanol (50 mL), hydrazine hydrate 90% (0.13 mL, 2.5 mmol) was added and the reaction mixture was refluxed for 6 h. After completion of the reaction, the reaction mixture was cooled and the precipitate that formed was filtered, dried and crystallized from dioxane to give the product; yield: 98%. 

*Method B*: To a solution of compound **3** (0.27 g, 1 mmol) in absolute ethanol (50 mL), hydrazine hydrate 90% (0.07 mL, 1.5 mmol) was added and the reaction mixture was refluxed for 6 h. After completion of the reaction, the reaction mixture was cooled and the precipitate that formed was filtered, dried and crystallized from dioxane to give the product; yield: 78%.

(Pale brown powder): mp over 300 °C; IR (KBr) ν_max_ in cm^−1^: 3386, 3260, 3172, 3124 (NH_2_, NH), 1605 (C=N); ^1^H-NMR (DMSO-*d*_6_): δ 4.58 (s, br, 4H, 2NH_2_; exchangeable with D_2_O), 7.42–7.60 (m, 3H, Ar-H), 8.60, 8.67 (2s, br, 2H, 2NH; exchangeable with D_2_O); ^13^C-NMR (DMSO-*d*_6_): δ 127.00, 129.44, 129.48, 131.26, 135.09, 137.07 (6Ar-C), 142.45, 146.57 (2C=N); MS (*m*/*z*), 196 (M^+^ − C_2_H_6_N_3_; 100%), 268 (M^+^; 43%), 269 (M^+^ + 1; 6%), 270 (M^+^ + 2; 43%). Anal. Calcd. for C_8_H_9_BrN_6_ (269.10): C, 35.71; H, 3.37; N, 31.23%. Found: C, 35.95; H, 3.50; N, 31.09%.

#### 3.1.8. 9-Bromoditetrazolo[1,5-*a*:5′,1′-*c*]quinoxaline (**11**)

*Method A*: A solution of sodium nitrite (0.07 g, 1 mmol) in the least amount of water was added dropwise to an ice-cold solution of compound **9** (0.27 g, 1 mmol) in acetic acid (30 mL) kept in an ice bath at 0 °C. The reaction mixture was allowed to stand overnight at room temperature, then it was poured into water (100 mL). The precipitate that formed was filtered, dried, crystallized from methanol and purified by a silica gel column chromatography (chloroform) to give the product; yield: 34%.

*Method B*: To a solution of compound **1** (0.28 g, 1 mmol) in absolute ethanol (50 mL), sodium azide (0.12 g, 2 mmol) was added and the reaction mixture was refluxed for two days. After completion of the reaction, the reaction mixture was cooled and the precipitate that formed was filtered, washed with a little amount of ethanol, dried and crystallized from methanol to give the product; yield: 85%.

(Brown powder): mp 229–231 °C; IR (KBr) ν_max_ in cm^−1^: 2155 (N_3_), 1608 (C=N); ^1^H-NMR (DMSO-*d*_6_): δ 8.26–9.01 (m, 3H, Ar-H); ^13^C-NMR (DMSO-*d*_6_): δ 119.61, 120.27, 122.00, 123.33, 123.59, 133.93 (6Ar-C), 140.37, 140.59 (2C=N); MS (*m*/*z*), 103 (M^+^ − C_6_H_3_N_8_, 100%), 290 (M^+^; 7%), 291 (M^+^ + 1; 1%), 292 (M^+^ + 2; 7%). Anal. Calcd. for C_8_H_3_BrN_8_ (291.07): C, 33.01; H, 1.04; N, 38.50%. Found: C, 32.87; H, 1.23; N, 38.69%.

#### 3.1.9. 7-Bromo-4,11-dimethyl-2,13-dihydrobis[1,2,4]triazino[4,3-*a*:3',4'-*c*]quinoxaline (**12**)

A mixture of compound **9** (0.27 g, 1 mmol) with chloroacetone (0.16 g, 2 mmol) in DMF (30 mL) and drops of glacial acetic acid (0.2 mL) was heated in 90 °C for 10 h. The solid that precipitated upon cooling was filtered off and crystallized from ethanol to give the product. Yield: 81%; (brown powder): mp over 300 °C; IR (KBr) ν_max_ in cm^−1^: 3310, 3291 (2NH), 2971, 2918 (aliphatic C-H), 1605 (C=N); ^1^H-NMR (DMSO-*d*_6_): δ 2.33 (s, 6H, 2CH_3_), 8.03–8.36 (m, 3H, Ar-H), 9.25 (s, 2H, C*H*-triazine), 10.03 (s, br, 2H, 2NH; exchangeable with D_2_O); ^13^C-NMR (DMSO-*d*_6_): δ 13.23, 13.87 (2CH_3_), 109.21 (2*C*H-triazine), 117.26, 123.65, 126.98, 127.43, 129.54, 131.54, 135.32, 138.00 (8Ar-C), 149.82, 149.93 (2C=N); MS (*m*/*z*), 344 (M^+^; 100%), 345 (M^+^ + 1; 23%), 346 (M^+^ + 2; 98%). Anal. Calcd. for C_14_H_13_BrN_6_ (345.20): C, 48.71; H, 3.80; N, 24.35%. Found: C, 48.52; H, 3.71; N, 24.54%.

#### 3.1.10. 9-Bromo-di-1,2,4-triazolo[1,5-*a*:5′,1′-*c*]quinoxaline (**13**)

A mixture of compound **9** (0.27 g, 1 mmol) and triethyl orthoformate (0.38 mL, 2.3 mmol) in ethanol (30 mL) and drops of acetic acid (0.1 mL) was heated in 90 °C for 10 h. The solid that precipitated upon cooling was filtered off and crystallized from acetic acid to give the product. Yield: 81%; (brownish powder): mp over 300 °C; IR (KBr) ν_max_ in cm^−1^: 1618 (C=N); ^1^H-NMR (DMSO-*d*_6_): δ 7.98–8.16 (m, 3H, Ar-H), 8.55 (s, 2H, 2C*H-*triazole); ^13^C-NMR (DMSO-*d*_6_): δ 110.6 (2*C*H-triazole), 121.36, 123.63, 127.29, 128.43, 134.42, 142.65 (6Ar-C), 147.81, 149.02 (2C=N); MS (*m*/*z*), 288 (M^+^; 100%), 289 (M^+^ + 1; 14%), 290 (M^+^ + 2; 96%). Anal. Calcd. for C_10_H_5_BrN_6_ (289.09): C, 41.55; H, 1.74; N, 29.07%. Found: C, 41.68; H, 1.81; N, 28.94%.

#### 3.1.11. 6-Bromo-3-(3,5-dimethyl-1*H*-pyrazol-1-yl)-2-hydrazinyl quinoxaline (**14**)

To a solution of compound **9** (0.27 g, 1 mmol) in absolute ethanol (50 mL), acetylacetone (0.11 mL, 1.1 mmol) and few drops of piperidine were added. The reaction mixture was refluxed for 15 h. After completion of the reaction, the reaction mixture was filtered, then the solution was allowed to stand overnight at room temperature. The precipitate that formed was filtered, dried and crystallized from ethanol to give the product. Yield: 50%; (orange powder): mp 164–166 °C; IR (KBr) ν_max_ in cm^−1^: 3448, 3277, 3191 (NH_2_, NH), 2976, 2924 (aliphatic C-H), 1601 (C=N); ^1^H-NMR (DMSO-*d*_6_): δ 2.01, 2.20 (2s, 6H, 2CH_3_), 4.93 (s, br, 2H, NH_2_; exchangeable with D_2_O), 6.05 (s, 1H, C*H*-pyrazole), 8.01–8.43 (m, 3H, Ar-H), 8.96 (s, br, 1H, NH; exchangeable with D_2_O); ^13^C NMR (DMSO-*d*_6_): δ 13.86, 13.94 (2CH_3_), 109.51 (*C*H-pyrazole), 117.21, 125.12, 125.41, 127.18, 134.65, 136.34, 138.85 (7Ar-C), 147.07, 149.82, 149.91 (3C=N); MS (*m*/z), 64 (M^+^ − C_9_H_11_BrN_5_; 100%), 301 (M^+^ − NHNH_2_; 14%), 302 (M^+^ +1 − NHNH_2_; 26%), 303 (M^+^ + 2 − NHNH_2_; 15%). Anal. Calcd. for C_13_H_13_BrN_6_ (333.19): C, 46.86; H, 3.93; N, 25.22%. Found: C, 47.02; H, 3.80; N, 25.04%.

#### 3.1.12. 6-Bromo-2,3-bis(3,5-dimethyl-1*H*-pyrazol-1-yl)quinoxaline (**15**)

*Method A*: To a solution of compound **9** (0.27 g, 1 mmol) in absolute ethanol (50 mL), acetylacetone (0.20 mL, 2 mmol) and few drops of piperidine were added. The reaction mixture was refluxed for 3 h. After completion of the reaction, the reaction mixture was filtered, then the solvent was evaporated under reduced pressure and residue obtained was dried and crystallized from chloroform to give the product; yield: 75%.

*Method B*: To a solution of compound **14** (0.33 g, 1 mmol) in absolute ethanol (50 mL), acetylacetone (0.10 mL, 1 mmol) and few drops of piperidine were added. The reaction mixture was refluxed for 6 h. After completion of the reaction, the reaction mixture was filtered, then the solvent was evaporated under reduced pressure and residue obtained was dried and crystallized from chloroform to give the product; yield: 62%.

(Yellow-brown powder): mp 96–98 °C; IR (KBr) ν_max_ in cm^−1^: 2955 (aliphatic C-H), 1600 (C=N); ^1^H-NMR (DMSO-*d*_6_): δ 2.12, 2.27, 2.28 (3s, 12H, 4CH_3_), 6.27 (s, 2H, 2C*H*-pyrazole), 7.43–7.68 (m, 3H, Ar-H); ^13^C-NMR (DMSO-*d*_6_): δ 11.47, 13.20, 21.66, 22.03 (4CH_3_), 104.37, 107.22 (2*C*H-pyrazole), 124.43, 130.21, 130.42, 134.47, 138.10, 139.85, 141.52, 141.66 (8Ar-C), 142.49, 142.94, 143.42, 149.27 (4C=N); MS (*m*/*z*), 396 (M^+^; 89%), 397 (M^+^ + 1; 100%), 398 (M^+^ + 2; 91%). Anal. Calcd. for C_18_H_17_BrN_6_ (397.27): C, 54.42; H, 4.31; N, 21.15%. Found: C, 54.31; H, 4.17; N, 21.04%.

#### 3.1.13. 6-Bromo-2,3-bis(3-amino-1*H*-pyrazol-5(4*H*)-one)quinoxaline (**16**)

To a warmed ethanolic sodium ethoxide solution [prepared by dissolving sodium metal (0.02 g, 1 mmol) in absolute ethanol (30 mL)] was added compound **9** (0.27 g, 1 mmol) and ethyl cyanoacetate (0.22 mL, 2 mmol). The mixture was stirred under reflux for 12 h, the reaction mixture was allowed to cool to room temperature, then poured into cold water (100 mL) and neutralized with acetic acid. The solid product was filtered off, washed with water, ethanol, dried and crystallized from ethanol to give the product. Yield: 35%; (pale brown crystals): mp 232–233 °C; IR (KBr) ν_max_ in cm^−1^: 3319, 3298 (NH_2_), 1695 (C=O), 1618 (C=N); ^1^H NMR (DMSO-*d*_6_): δ 3.95 (s, 4H, 2C*H*_2_-pyrazole), 8.08–8.24 (m, 3H, Ar-H), 11.2 (s, br, 4H, 2NH_2_; exchangeable with D_2_O); ^13^C-NMR (DMSO-*d*_6_): δ 75.32 (2*C*H_2_-pyrazole), 121.98, 126.14, 128,10, 130.32, 134.65, 142.68 (6Ar-C), 153.23, 153.28, 159.62, 159.69 (4C=N), 169.06, 169.12 (2C=O); MS (*m*/*z*), 402 (M^+^; 64%), 403 (M^+^ + 1; 11%), 404 (M^+^ + 2; 65%). Anal. Calcd. for C_14_H_11_BrN_8_O_2_(403.19): C, 41.70; H, 2.75; N, 27.79%. Found: C, 41.82; H, 2.62; N, 27.94%.

#### 3.1.14. 6-Bromo-2,3-bis(3-methyl-1*H*-pyrazol-5(4*H*)-one)quinoxaline (**17**)

A solution of compound **9** (0.27 g, 1 mmol) and ethyl acetoacetate (0.26 mL, 2 mmol) in sodium ethoxide solution [prepared by dissolving sodium metal (0.02 g, 1 mmol) in absolute ethanol (30 mL)] was heated under reflux with stirring for 4 h. The reaction mixture was allowed to cool and poured into cold water (100 mL) and neutralized by acetic acid, whereby a solid was precipitated, which was filtered off and crystallized from chloroform. Yield: 43%; (pale brown crystals): mp 152–154 °C; IR (KBr) ν_max_ in cm^−1^: 1698 (C=O), 1621 (C=N); ^1^H-NMR (DMSO*-d*_6_): δ 2.33 (s, 6H, 2CH_3_), 3.84 (s, 4H, 2C*H*_2_-pyrazole), 7.89–8.06 (m, 3H, Ar-H); ^13^C-NMR (DMSO-*d*_6_): δ 16.31, 16.38 (2CH_3_), 71.25 (2*C*H_2_-pyrazole), 123.74, 126.87, 128.53, 129.98, 131.34, 141.18 (6Ar-C), 152.36, 152.41, 158.12, 158.19 (4C=N), 169.10, 169.21 (2C=O); MS (*m*/*z*), 400 (M^+^; 79%), 401 (M^+^ +1; 20%), 402 (M^+^ + 2; 80%). Anal. Calcd. for C_16_H_13_BrN_6_O_2_(401.22): C, 47.90; H, 3.27; N, 20.95%. Found: C, 48.02; H, 3.16; N, 21.13%.

### 3.2. Pharmacological Evaluation

#### 3.2.1. Anticancer Activity

##### Cell Cultures

The newly synthesized compounds were evaluated *in vitro* against three human cancer cell lines; which are MCF-7 (breast adenocarcinoma), NCI-H460 (non-small cell lung cancer) and SF-268 (CNS cancer), and WI 38 (normal fibroblast cells) were used in this study. MCF-7 was obtained from the European Collection of Cell Cultures (ECACC, Salisbury, UK) but NCI-H460, SF-268 and WI 38 were kindly provided by the National Cancer Institute (NCI, Cairo, Egypt). They grow as monolayer and routinely maintained RPMI-1640 medium supplemented with 5% heat inactivated fetal bovine serum (FBS), 2 mM glutamine and antibiotics (penicillin 100 U/mL, streptomycin 100 µg/mL), at 37 °C in a humidified atmosphere containing 5% CO_2_. Exponentially growing cells were obtained by plating 1.5 × 10^5^ cells/mL for MCF-7 and SF-268, and 0.75 × 10^4^ cells/mL for NCI-H460 followed by 24 h of incubation. The effect of the vehicle solvent (DMSO) on the growth of these cell lines was evaluated in all experiments by exposing untreated control cells to the maximum concentration (0.5%) of DMSO used in each assay.

##### Cancer Cell Growth Assay

The effect of compounds on the *in vitro* growth of human tumor cell lines were evaluated according to the procedure adopted by the National Cancer Institute (NCI, Austin, TX, USA) in the “*In vitro* Anticancer Drug Discovery Screen” that uses the protein-binding dye sulforhodamine B (SRB) to assess cell growth [39]. In the assay protocol, all cells were incubated at 37 °C under humidified atmosphere containing 5% CO_2_. Briefly, exponentially cells growing in 96-well plates were then exposed for 48 h to five serial concentrations of each compound, starting from a maximum concentration of 150 μg/mL. Following this exposure period adherent cells were fixed, washed and stained. The bound stain was solubilized and the absorbance was measured at 492 nm in a plate reader (Bio-Tek Instruments Inc., Power wave XS, Winston, NC, USA). For each test compound and cell line, a dose response curve was obtained and the inhibitory concentration of 50% (IC_50_), corresponding to the concentration of the compounds that inhibited 50% of the net cell growth was calculated as described elsewhere [40]. Doxorubicin was used as a positive control and tested in the same manner.

#### 3.2.2. Antimicrobial Activity

##### Preparation of Microbial Suspensions

Antimicrobial activities were carried out against highly pathogenic strains; three strains of Gram positive bacteria (*Bacillus subtilis, Staphylococcus aureus* and *Streptococcus faecalis*), four strains of Gram negative bacteria (*Escherichia coli*, *Neisseria gonorrhoeae*, *Pseudomonas aeruginosa* and *Salmonella typhimurium*) and three fungi strains (*Aspergillus flavus*, *Aspergillus fumigatus* and *Candida albicans*) isolated from primary agar plates. Antimicrobial activity of the tested samples was determined using a modified Kriby-Bauer disk diffusion method [41]. Briefly, 100 µL of the test bacteria/fungi were grown in 10 mL of fresh media until they reached a count approximately 10^8^ cells/mL for bacteria or 10^5^ cells/mL for fungi [42]. 100 µL of microbial suspension was spread onto agar plates corresponding to the broth in which they were maintained. The inoculated plates of bacterial strains were incubated at 35–37 °C for 24–48 h, while the inoculated plates of fungal strains such as *A. flavus* and *A. fumigatus* were incubated at 25 °C for 48 h and yeast as *C. albicans* was incubated at 30 °C for 24–48 h [41].

##### Determination of Antimicrobial Activity by Disk Diffusion Method

The agar disk diffusion (qualitative) method was used in this investigation for determination of the preliminary antibacterial and antifungal activities. The agar used was Mueller-Hinton agar plates that are rigorously tested for composition and pH. Further the depth of the agar in the plate is a factor to be considered in the disk diffusion method.

Reference drugs: ampicillin was used as a standard antibacterial drug for Gram positive bacteria, gentamicin as a standard antibacterial drug for Gram negative bacteria and amphotericin B as antifungal standard drug. In addition, standard filter disks impregnated with 10 µL of DMSO as solvent which showed no zone of inhibition and acts as a negative control. 

Bacterial and fungal strains were spread onto the surface of the agar plates using sterile cotton swabs. For evaluation of antibacterial activities, blank paper disks (Schleicher & Schuell, Alicante, Spain) with a diameter of 8.0 mm were saturated with 10 μL of tested concentration of the stock solutions. Disks were dried and then placed onto inoculated agar plates. The plates of bacterial strains were reincubated at 35–37 °C for 24–48 h, while the plates of fungal strains such as *A. flavus* and *A. fumigatus* were reincubated at 25 °C for 48 h and yeast as *C. albicans* was reincubated at 30 °C for 24–48 h as mentioned above. After incubation, plates were observed for antimicrobial activities by determining the diameters of inhibition zones (IZ) of bacterial or fungal growth around the disks of the samples in (mm) by slipping calipers of the Clinical and Laboratory Standared Institute CLSI [42]. For an accurate analysis, tests were run in triplicate for each strain to avoid any error as described in Table 2.

Disk diffusion method for filamentous fungi testing by using approved standard method (M02-A11) developed by the CLSI for evaluating the susceptibilities of filamentous fungi to antifungal agents [43]. Disk diffusion method for yeasts developed by using approved standard method (M44-A2) by the CLSI [44]. Agar-based methods such as disk diffusion can be good alternatives because they are simpler and faster than broth-based methods [45].

##### Determination of Minimum Inhibitory Concentration (MIC)

In this study, MIC (quantitative method) was used for evaluation of the antimicrobial activity of tested compounds. Screening was evaluated *in vitro* using the Broth dilution method according to CLSI [45]. All the bacteria were incubated and activated at 30 °C for 24 h inoculation into nutrient broth and the fungi were incubated in malt extract broth for 48 h. The compounds were dissolved in DMSO and then diluted using cautiously adjusted Mueller-Hinton broth. Two-fold serial concentrations dilution method (0.98, 1.95, 3.9, 7.81, 15.63, 31.25, 62.5, 125 µg/mL) of the some compounds were employed to determine the (MIC). In each case triplicate tests were performed and the average was taken as the final reading. The tubes were then inoculated with the test organisms, grown in their suitable broth at 37 °C for 24 h for tested microorganisms (1 × 10^8^ CFU/mL for bacteria and 1 × 10^6^ CFU/mL of fungi), each 5 mL received 0.1 mL of the above inoculum and incubated at 37 °C for 24 h. The lowest concentration showing no growth was taken as the minimum inhibitory concentration (MIC); shown in Table 3.

## 4. Conclusions

The objective of this work was to design and synthesize new compounds with anticancer and antimicrobial properties at the same time. The anticancer evaluation of the newly synthesized quinoxaline derivatives showed that among the tested compounds 8-bromo-4-chlorotetrazolo[1,5-*a*]quinoxaline (**4**), 8-bromo-4-(piperidin-1-yl)tetrazolo[1,5-*a*]quinoxaline (**5a**), 8-bromo-4-(morpholin-4-yl)tetrazolo[1,5-*a*]quinoxaline (**5b**) and 9-bromoditetrazolo[1,5-*a*:5',1'-*c*]quinoxaline (**11**) showed the best result, exhibiting the highest inhibitory effects towards the three tumor cell lines, which were higher than that of the reference doxorubicin and these compounds were non-cytotoxic on the normal cells (IC_50_ values >100 μg/mL). In addition, compounds **4**, **5a**, **5b**, **9** and **11**–**13** exhibited the highest degrees of inhibition against the strains of Gram positive and negative bacteria. Such high activity of **4**, **5a**, **5b** and **11** was may be due to the presence of a tetrazolo ring combined with a quinoxaline moiety. 
